# Using the autologous innominate vein as a substitute for pulmonary arteries in a patient with pulmonary atresia and absent pulmonary arteries

**DOI:** 10.1186/s13019-021-01489-9

**Published:** 2021-04-15

**Authors:** Ahmad Ali Amirghofran, Kamran Jamshidi, Mohammadreza Edraki, Hamid Amoozgar, Farah Peiravian, Elahe Nirooei

**Affiliations:** 1 Shiraz University of Medical Sciences, Shiraz, Iran; 2 Islamic Azad University, Kazerun, Iran

**Keywords:** Innominate vein, Case report, Absent pulmonary artery, Pulmonary atresia, Autologous

## Abstract

**Background:**

Repair of the absence of the whole or major parts of pulmonary arteries is a challenge, and the choice of conduit material to reconstruct the pulmonary arteries is under dispute. We used the autologous innominate vein to construct pulmonary arteries.

**Case presentation l:**

We present a novel technique using the autologous innominate vein as a free graft in a 6-month-old infant with pulmonary atresia and absence of central pulmonary arteries. Double ductus arteriosus were the only source of perfusion of the lungs. The innominate vein was substituted for the central pulmonary artery between the two lung hila. Total repair by using Contegra graft was performed 9 months later. The patient has been followed for 5 years.

**Conclusions:**

The autologous innominate vein could be used as inter-hilar pulmonary arteries with no calcification and fibrosis in 5-year follow-up.

## Introduction

Repair of pulmonary atresia when there is a complete absence of central pulmonary arteries is quite challenging. A “tubal structure” is required to restore the continuity and confluence of lung hila, which will then be connected to the right ventricle in the final corrective surgery.

Even though different types of biologic or non-biologic materials have been used for this purpose, none have ideal conduit characteristics. Lack of growth potential, calcification, thrombosis, peeling formation by intimal hyperplasia, shrinkage, fibrosis, and stricture are the major disadvantages of tubes made of different materials such as synthetic materials, homografts, xenografts, or pericardium [1]. This paper introduces the first deployment of the innominate vein as a free native substitute for absent pulmonary arteries in pulmonary atresia and its 5-year follow-up result.

## Case presentation

**Fig. 1:**
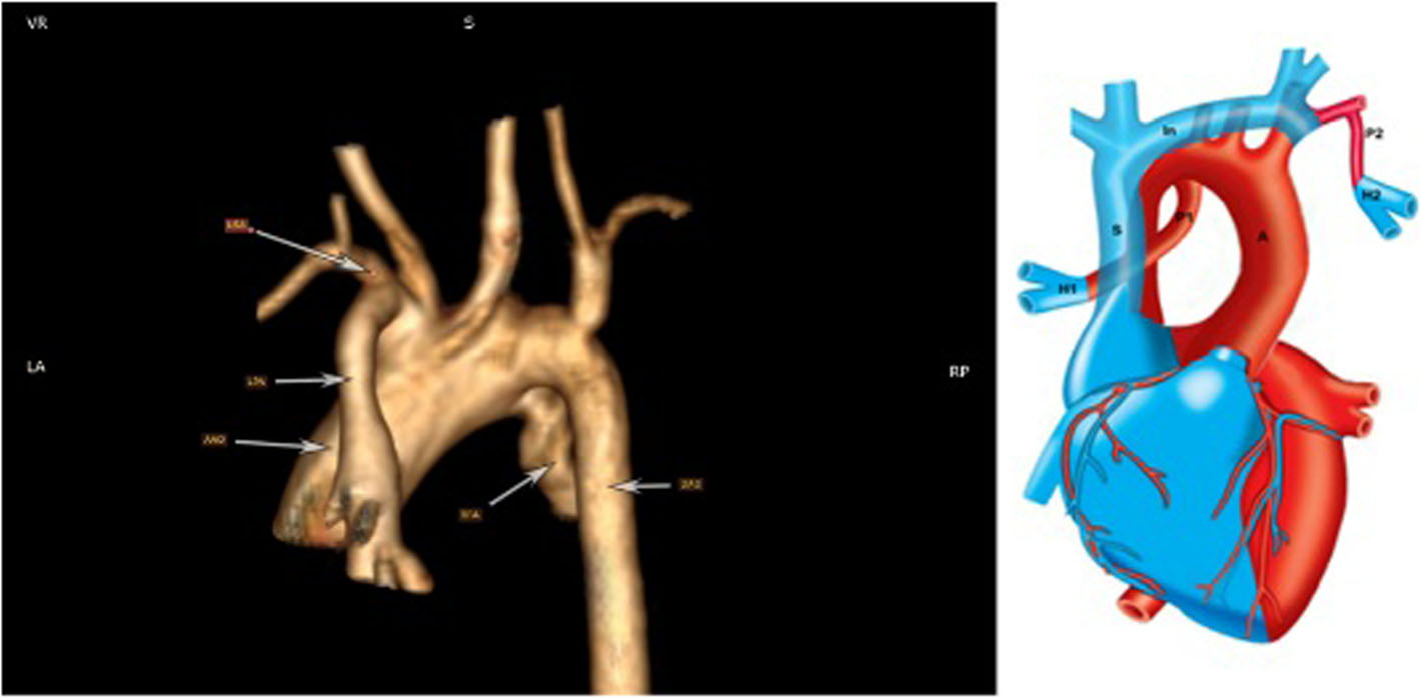
CT angiography and schematic picture show the anatomy before the surgery. A, aorta; AAO, ascending aorta; DAO, descending aorta; H1, right hilum; H2, left hilum; In, innominate; LPA, left pulmonary artery; LSA, left subclavian artery; P1, right patent ductus arteriosus; P2, left patent ductus arteriosus; RPA, right pulmonary artery; S, superior vena cava

Pulmonary atresia, double outlet right ventricle, ventricular septal defect, and complete absence of major pulmonary arteries were diagnosed in a 6 m/o infant by echocardiography and CT-angiography (Fig. 1a, b). Both lung hila were connected to the aorta through two separate ducti arteriosus. No large collateral existed, and the gap between the two lung hila was approximately 5 cm.

The first stage operation was performed to reconstruct the pulmonary arteries through a median sternotomy on normothermic beating heart cardio-pulmonary bypass.

**Fig. 2:**
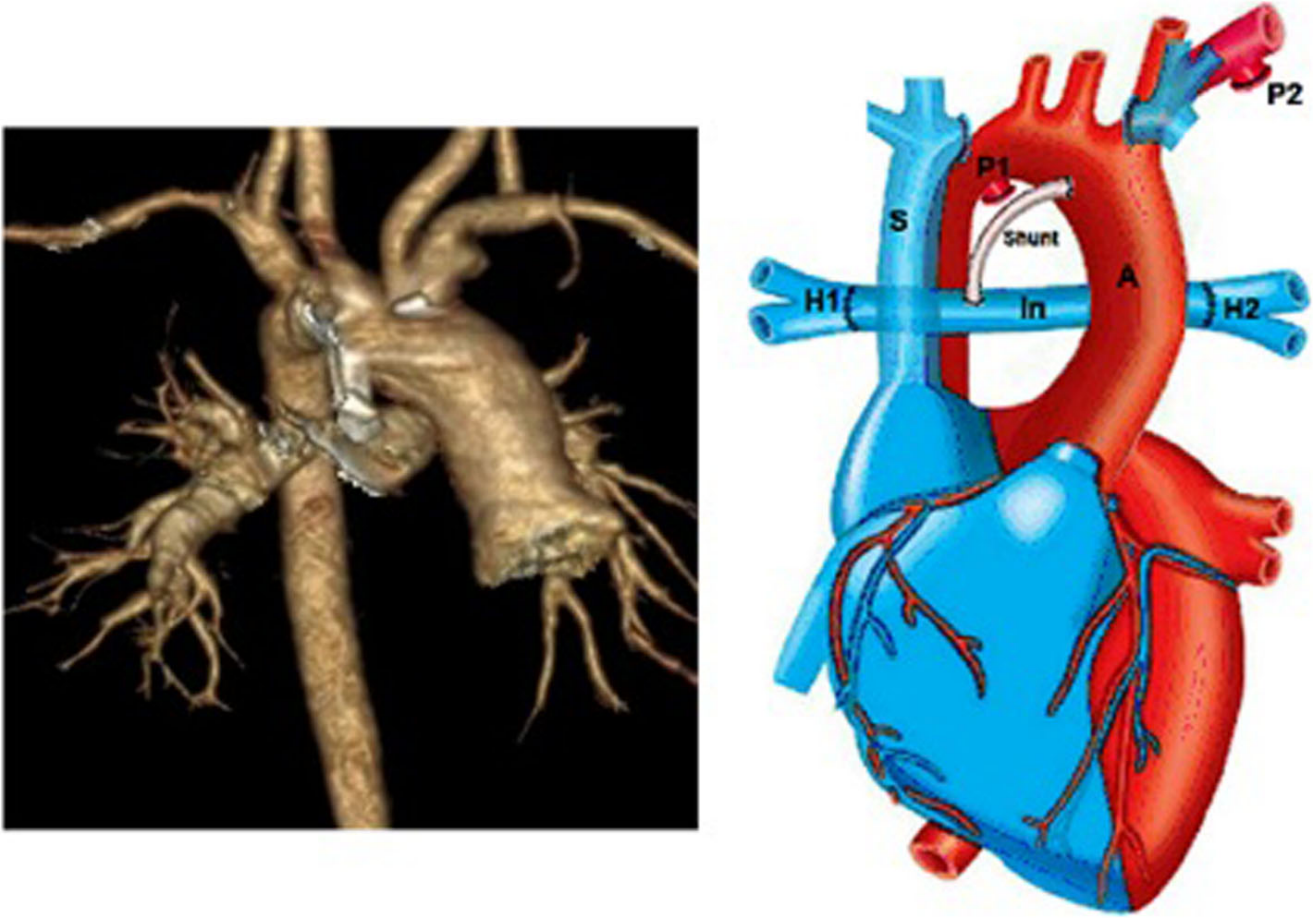
CT angiography and schematic picture after the innominate vein interposition between the right and left hila. A, aorta; H1, right hilum; H2, left hilum; In, innominate; P1, right patent ductus arteriosus; P2, left patent ductus arteriosus; S, superior vena cava

To ensure that there are sufficient venous connections between the left and right upper body venous systems, the innominate vein was temporarily clamped for 5 min before cardio-pulmonary bypass. The venous pressure in the left side of clamping site was measured directly to check the increase not to exceed 5 mmHg which was considered as the significant pressure change. Then, both ducti arteriosus and their connection to the lung hila were dissected and exposed as well as the innominate vein in its entire course, and the hilar areas were cleared of any suspected residual ductal tissue. The innominate vein was transected at its connection to the left jugular and subclavian vein and connected to the left hilar opening with a wide anastomosis. Then it was transected in the right side at its junction with the superior vena cava, passed behind the ascending aorta, and anastomosed to the right hilum. A 5 mm shunt interposed between the ascending aorta and the conduit near the right hilum (Fig. 2a, b).

The cardio-pulmonary bypass was discontinued; pressures were measured again, which revealed a 5 mm difference between left and right venous systems.

The patient tolerated the procedure well with no facial or arm edema.

During the next 6 months, blood oxygen saturation gradually decreased. Echocardiographic and catheterization evaluations revealed shunt narrowing. Therefore, shunt stenting was performed, which resulted in an early increase in oxygen saturation. After 3 months, a shunt re-stenosis confirmed by CT angiography occurred. Besides, moderate stenosis of the innominate vein anastomosis to the right hilum was detected (Fig. 2a). Hence, considering all conditions, we decided to proceed to a total repair 9 months after the initial pulmonary artery reconstruction.

The operation was done through a median sternotomy on cardiopulmonary bypass and cardioplegic arrest. The quality of the innominate vein tissue was observed. The wall was thicker than the original, but the tissue was completely flexible, stretchable, and soft with no sign of fibrosis, calcification, or internal peeling formation. However, the internal lumen seemed to be relatively narrow despite elastic and stretchable wall tissue. The ventricular septal defect was closed after enlargement to overcome the severe overriding of the aorta, and an 18 mm Contegra graft (Medtronic Inc., Minneapolis, MN) was used to establish continuity between the right ventricle and the innominate vein. The Contegra graft inexorably crossed anteriorly towards the right side because the extremely large aorta was completely deviated to the left side.

**Fig. 3:**
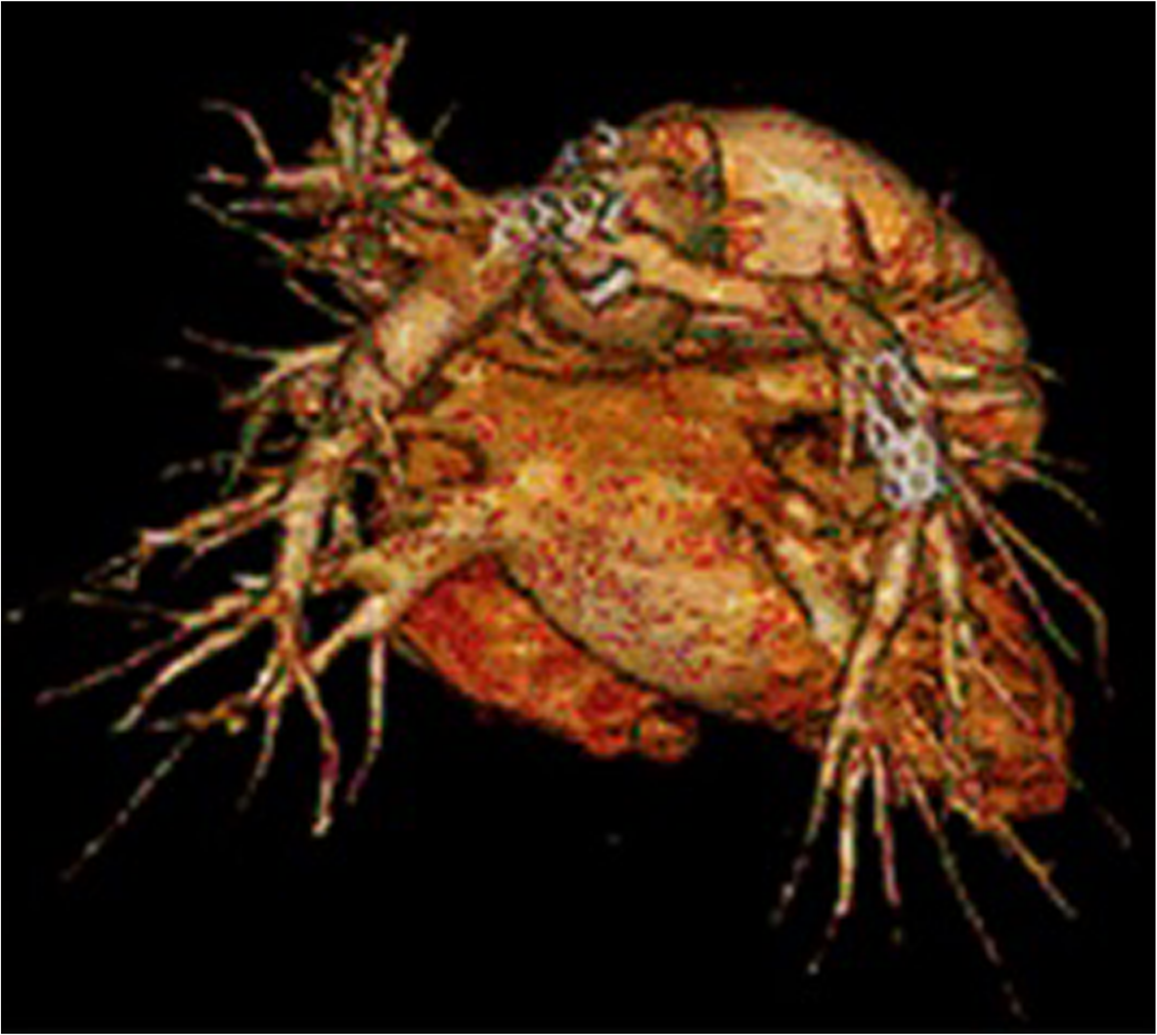
CT Angiography 3 years after the total repair by Contegra graft between the right ventricle and the innominate vein. The distal Contegra graft stenosis was stented. Please note the migration of the second stent from the left pulmonary artery into the distal left lung hilum

The patient has generally been well since the final operation performed 4 years ago. A gradual, moderate increase in the gradient across pulmonary arteries was demonstrated in regular follow-up echocardiography. CT angiography confirmed a moderate narrowing of the distal anastomotic site of the Contegra graft on both sides. Consequently, bilateral stenting was performed after 3 years, followed by a re-dilatation 1 year later (Fig. 3). The patient is now in NYHA class I and good condition.

## Discussion

The ideal conduit required to be used as a substitute for pulmonary arteries should be an autologous tissue with the possibility of growth, resistance to thrombosis, and lifelong lasting. However, such a conduit is not identified, and most of the available options, including Gortex grafts, pericardial tubes, and homografts, neither grow nor are amenable to catheter dilatation due to fixed-size synthetic material or organic tissue fibro-calcification and therefore cannot compensate for the child growth. On this prospect, we speculated that soft native tissue, even if it does not grow, may have the advantage of distensibility by catheter-based interventions. The innominate vein normally has a length and caliber similar to the inter-hilar pulmonary arteries. The extensive venous interconnections will compensate for venous drainage of the left side of the brain and left upper extremity precluding high venous pressure and edema. As described, we did test the venous collateral capability in this case; however, innominate vein transection has been proven to be safe in aortic aneurysm surgery without any routine pressure test measurements [2]. The whole length of the innominate vein may be removed and interposed between hila of the right and left lungs, which can then be connected to the right ventricle by a homograft or xenograft in the next stage.

We observed very good tissue quality resembling the normal pulmonary artery wall in terms of thickness and softness, and elasticity in the second operation. Although some narrowing could be demonstrated before the operation, no fibrosis or calcification could be seen operatively, indicating that the narrowing was more likely a dynamic process than a structural change. This assumption became more pronounced when we noticed that one stent placed and dilated in the mid part of the left side of the conduit had moved to the distal intrapulmonary branches, which demonstrated that the stenosis has possibly been very soft and flexible rather than rigid and structural. Even though we cannot confirm the growth of this novel conduit and we have observed gradual narrowing in some parts of the innominate conduit, we think that it has the very good advantage of possible staged dilatation by the catheter-based intervention. Our experience showed that the balloon-expandable stents could be placed easily in this conduit with low-pressure dilatation, and repeated re-dilatation can be performed due to tissue elasticity until it reaches the final sufficient adult size.

## Conclusion

The autologous innominate vein could be used as the inter-hilar pulmonary arteries in patients with pulmonary atresia and absent pulmonary arteries. Fibrotic change and calcification were not seen in our patient after 5 years, and interventional stent insertion could be performed with no difficulties.

## Data Availability

we state that the data used are available, and data sharing is allowed.

## Abbreviations

CT: Computerized tomography
NYHA: New York Heart Association
